# C-Type Natriuretic Peptide Ameliorates Lipopolysaccharide-Induced Cardiac Dysfunction in Rats with Pulmonary Arterial Hypertension

**DOI:** 10.1155/2018/2813025

**Published:** 2018-12-20

**Authors:** Xiaowei Gao, Maoen Zhu, Yanan Cao, Yue Yang, Zhi Ye, Fan Zhang, Qulian Guo, Yonggang Peng, E. Wang

**Affiliations:** ^1^Department of Anesthesiology, Xiangya Hospital, Central South University, Changsha, Hunan, China; ^2^Department of Anesthesiology, University of Florida College of Medicine, Gainesville, Florida, USA

## Abstract

Lipopolysaccharide induces rapid deterioration of cardiac function in rats with pulmonary arterial hypertension. It was desired to investigate if this cardiac dysfunction could be treated by C-type natriuretic peptide. Rat pulmonary arterial hypertension was induced by intraperitoneal injection of monocrotaline. Hemodynamics and cardiac function were measured by pressure-volume (P-V) catheter before and after the rats were treated with lipopolysaccharide and C-type natriuretic peptide. Cyclic guanosine 3′,5′-monophosphate (cGMP) level was determined by enzyme-linked immunosorbent assay analysis. After the rats were injected with low-dose lipopolysaccharide, they experienced left ventricle systolic function deterioration. Administration of C-type natriuretic peptide improved hemodynamics and left ventricle systolic function. cGMP level was elevated after C-type natriuretic peptide treatment. C-type natriuretic peptide could ameliorate lipopolysaccharide-induced cardiac dysfunction and restore hemodynamic deterioration in rats with pulmonary arterial hypertension.

## 1. Introduction

Pulmonary arterial hypertension (PAH) leads to profound stress of the right ventricle (RV) and to right heart failure [1]. Ventricular interaction during the diastolic phase, in which the filling of one ventricle is influenced by the volume or pressure of another ventricle, may be aggravated by pulmonary hypertension and volume overload [2]. It has been reported that sepsis can exacerbate pulmonary hypertension and increase RV afterload [3]. In our previous study, we demonstrated that 1 mg/kg LPS could induce acute right heart failure in rats with PAH [4]. Clinically, patients with PAH suffering from inflammation are more prone to severe complications of multiple organ system failure, including cardiac dysfunction, respiratory distress, and even death. It is still unclear whether deterioration of cardiac function in patients who have PAH with inflammation can be reversed.

C-type natriuretic peptide (CNP) is secreted by vascular endothelial cells [5]. It exhibits natriuretic, diuretic, vasorelaxant, and anti-hypertrophic properties and may have compensatory effects in the presence of heart failure, hypertension, coronary artery disease, and ventricular hypertrophy [6]. The hypothesis of our study is that CNP treatment could improve the cardiac function of pulmonary arterial hypertensive rats in circumstances of lipopolysaccharide- (LPS-) induced inflammation.

## 2. Materials and Methods

All experimental procedures and protocols in this research were approved by the Ethics Committee of Xiangya Hospital of Central South University (201303311). All operations were carried out under anesthesia and the suffering of animals was minimized as much as possible.

### 2.1. PAH Model

Adult male Sprague-Dawley rats (6–8 weeks old) weighing 300 to 350 g were treated with monocrotaline (55 mg/kg, intraperitoneal injection, Crotaline C2401, Sigma-Aldrich, Buchs, Switzerland) to induce PAH [7]. Control rats were injected with equal volumes of normal saline. All rats were fed and housed in conditions of controlled temperature and humidity with a 24-hour circadian rhythm and were given standard food and tap water.

### 2.2. Morphological Study

Rat hearts were dissected after rats were euthanized. RV hypertrophy was indicated by the Fulton index, which is the ratio of the weight of the RV wall to the left ventricular (LV) wall and the ventricular septum (S) (RV/[LV + S]) [8]. The right lungs and myocardium of the rat's RVs were fixed with 10% paraformaldehyde and embedded in paraffin. The final step was to section the tissue of the right lungs and myocardium for hematoxylin and eosin staining. The blood drawn from the hearts was centrifuged, and the plasma was preserved at −20°C for later biochemical measurements and analysis.

### 2.3. Hemodynamics and Cardiac Function Measurements

Four weeks after monocrotaline injection, all rats were anesthetized by an injection of ketamine (50 mg/kg). The airway was secured by tracheostomy and cannula. Rats were immediately ventilated with a constant volume-cycled animal ventilator (Model 683, Harvard Apparatus, Holliston, MA). Parameters of respiration were set as tidal volume, 5 mL/kg, respiratory rate, 70 to 80 breaths/min, and an FiO_2_ of 100%; these were adjusted according to arterial blood gas analysis. Anesthesia was maintained by 1% to 2% (v/v) sevoflurane inhalation. All rats were placed on a homoeothermic blanket to keep their body temperature at 37°C. Each rat's left saphenous vein was catheterized with 24-gauge cannula to infuse a crystalloid solution at 10 mL/kg/h. Each rat's right carotid artery was catheterized with a 1.9-F conductance pressure-volume (P-V) catheter (Model SPR-838, Millar Instruments Inc., Houston, TX) to measure LV function without sternotomy. Functional parameters were monitored and recorded with a PowerLab system (ADInstruments, Bella Vista, New South Wales, Australia). Correct position was calibrated by phase and magnitude signals as well as by visualization of the P-V loops and the ability to modify the P-V loops when altering preload by transient inferior vena cava occlusion. When LV function measurement was finished, the right external jugular vein was catheterized with a PE 50 catheter (Hongye Plastics Co. Ltd, Shanghai, China) to measure RV systolic pressure. All data were then analyzed using LabChart 7 software (ADInstruments). Each group included 12 rats.

The systolic function indices derived by P-V loops are less influenced by loading conditions and cardiac mass. From P-V recordings, we acquired heart rate, stroke volume, cardiac output, stroke work, ejection fraction, the rate (dP/dt max) of LV pressure rise in early systole, and time constant of relaxation (Tau). The software calculated parallel conductance volume from the shift of P-V relations, and this was used for the correction of the cardiac volume. The conductance system was calibrated with saline according to the instruction manual.

### 2.4. LPS and CNP Experiments

In the phase I experiment, normal rats and PAH rats were randomly assigned to the control group (normal saline) or the LPS group (1 mg/kg, Sigma, St. Louis, MO) injection. Hemodynamics and cardiac function were measured, respectively.

**Figure 1:**
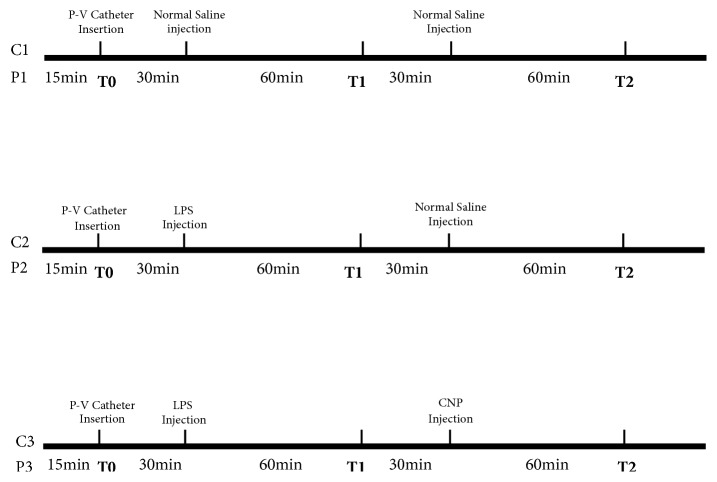
The protocol of phase II experiment. C1, C2, and C3 are groups of normal rats. P1, P2, and P3 are groups of pulmonary artery hypertension rats. T0: baseline values of hemodynamic data were recorded. T1: hemodynamic data were recorded after normal saline or LPS injection. T2: hemodynamic data were recorded after normal saline or CNP injection.

In phase II of the experiment, PAH rats were randomly divided into groups P1, P2, and P3. Normal rats were randomly divided into groups C1, C2, and C3. Each group included 10 to 12 rats. Baseline values of hemodynamic data were recorded when hemodynamics stabilized at T0. Rats in groups P1 and C1 received normal saline by intraperitoneal injection as a control. Rats in groups P2 and P3, C2, and C3 received LPS by intraperitoneal injection. After 1 hour, the parameters measured by P-V catheter were collected at T1. Rats in groups P1 and P2, C1, and C2 then received a normal saline infusion and the rats in group P3 and C3 were treated with CNP (CNP-53, The Peptide Institute, Osaka, Japan). CNP was given with a 2 *μ*g/kg loading dose plus a 0.4 *μ*g/kg/min infusion for 20 minutes [9]. Data were then recorded after 1 hour at T2. The protocol of phase II experiment was showed in Figure 1.

### 2.5. Enzyme-Linked Immunosorbent Assay (ELISA) for Cyclic Guanosine 3′,5′-Monophosphate (cGMP)

Blood samples were collected from each control group and PAH group for use in determining the effects of CNP on plasma levels of cGMP in PAH rats with LPS treatment. Plasma cGMP concentrations were detected with the specific commercial radioimmunoassay of a cyclic GMP ELISA kit (Elabscience Biotechnology Inc., Hubei, China). The protocol of ELISA for cGMP was performed according to the kit's instructions: add 50 *μ*L of standard or sample to each well and then immediately add 50 *μ*L of biotinylated detection Ab to each well; incubate for 45 minutes at 37°C. After this steps, aspirate and wash three times. Add 100 *μ*L of horseradish peroxidase conjugate to each well and then incubate for 30 minutes at 37°C. Aspirate and wash this five times. Add 90 *μ*L of substrate reagent and then incubate for 15 minutes at 37°C and add 50 *μ*L of stop solution. Immediately read the results at 450 nm with an EL×800 Automated Microplate Reader (BioTek Instruments, Inc., Winooski, VT).

### 2.6. Statistical Analysis

Statistical analysis was performed with GraphPad Prism (version 6.01, GraphPad Software, San Diego, CA). The data were expressed as mean ± standard deviation. For hemodynamic and LV P-V parameters, the two-way analysis of variance was used to evaluate the difference over time. Significance was established as* P* < 0.05.

## 3. Results

### 3.1. PAH Rat Model and Pathological Change

Table 1 shows morphological data for the hearts of rats with or without monocrotaline treatment. Body weight was statistically different between the two groups (*P* < 0.05) but Heart weight was not statistically different between (*P* > 0.05). RV weights of rats in the PAH group were significantly higher than those of rats in the normal saline group (*P* < 0.05). LV + S weights of rats in the PAH group were significantly higher than those of rats in the normal saline group (*P* < 0.05). The ratio of heart weight to body weight was statistically different between the two groups (*P* < 0.05). The RV/(LV + S) weight ratio was increased significantly in the PAH group (*P* < 0.05). The index of mean arterial pulmonary arterial pressure for rats in the PAH group was elevated significantly (*P* < 0.05).

**Figure 2:**
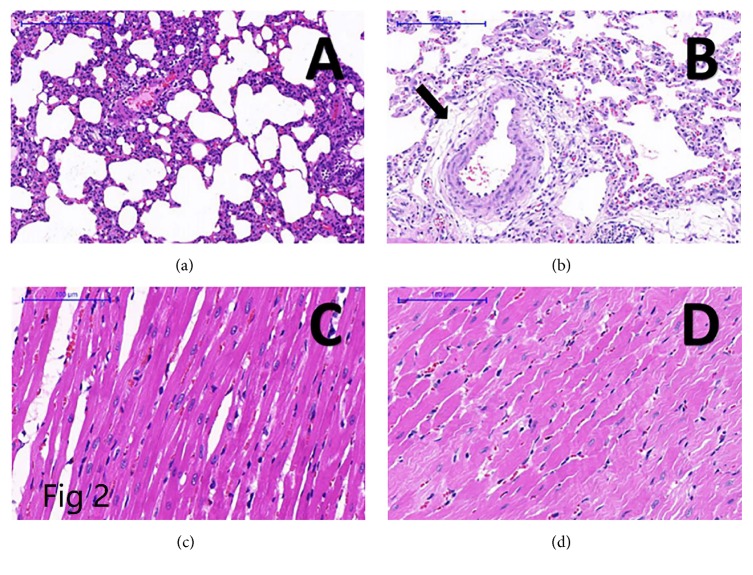
Hematoxylin and eosin staining of the lung and right ventricle from a normal rat ((a) and (c)) and a PAH rat ((b) and (d)). The proliferation of smooth muscle cells, collagen fiber deposition in the vessel wall, thickening, and dense inflammatory infiltration were observed around the arterioles in PAH rats. Some of the right ventricular myocytes were fractured from cytoplasm swelling in PAH rats.

The proliferation of smooth muscle cells, collagen fiber deposition in the vessel walls, thickening, and dense inflammatory infiltration around the arterioles were observed in hematoxylin and eosin staining of the lungs of PAH rats. In addition, in PAH rats, some of the RV myocytes were fractured from cytoplasm swelling (Figure 2).

### 3.2. LPS Injection Induced Deterioration of Cardiac Function in PAH Rats

The effects of LPS on the hemodynamics and cardiac function of normal and PAH rats are shown in Table 2. Low doses of LPS did not significantly affect normal rats. Heart rate, end systolic volume, end diastolic volume, end systolic pressure, cardiac output, ejection fraction, dP/dt_max⁡_, and stroke work of PAH rats significantly decreased after LPS treatment, whereas Tau all increased.

### 3.3. Effect of CNP on LPS-Treated PAH Rats

As shown in Table 3, after being treated with LPS, PAH rats were found to have significantly decreased heart rate, end diastolic volume, end systolic pressure, stroke volume, cardiac output, dP/dt_max⁡_, stroke work, and Tau when compared with baseline. The changes for groups P2 and P3 were consistent at T1. After rats in group P2 were treated with normal saline sequentially, the parameters depressed at T2 as much as at T1. However, for rats in group P3, hemodynamics and cardiac function at T2 improved significantly after CNP infusion when compared with T0 or group P2. These results indicate that CNP has a positive inotropic effect on LV contractile performance. CNP infusion can restore cardiac function in PAH rats with inflammation.

### 3.4. Plasma Levels of cGMP after CNP Administration

**Figure 3:**
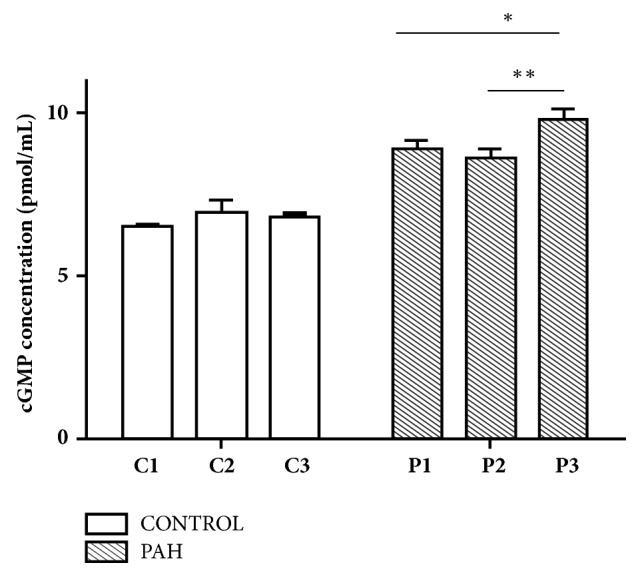
The cGMP concentration of plasma in normal and PAH rats with or without LPS and CNP treatment. Data are presented as mean ± SD. C1: control group treated with normal saline. C2: control group treated with LPS. C3: control group treated with LPS and CNP. P1: PAH group treated with normal saline. P2, PAH group treated with LPS. P3: PAH group treated with LPS and CNP. The values of cGMP were significantly increased in P3 group when compared with P1 and P2 groups.

Plasma cGMP levels for each group are shown in Figure 3. There was no significant change in the control group, even after treatment with LPS and CNP. However, the rats in the PAH group had significantly increased plasma cGMP levels after treatment with LPS and sequential CNP.

## 4. Discussion

Monocrotaline was widely used for developing rat models of PAH, which resulted from injury to pulmonary endothelial cells [10, 11]. This model is more likely to lead to the development of PAH and exhibits more serious hemodynamic and histopathological changes than Sugen 5416/chronic hyoxia rats [12, 13]. The morphological images demonstrate that RV hypertrophy and pulmonary endothelial injury appear in this PAH model. The PAH rats show an elevation of mean pulmonary arterial pressure and right-to-left ratio of ventricular pressure. These results suggest that monocrotaline successfully induces PAH, as previous experiments have shown [7, 8].

Heart failure in the PAH model was caused by LPS-induced inflammation. In our study, the LPS injection appears to mimic acute inflammation that makes patients with PAH more vulnerable to acute heart failure. We compared LV cardiac function using a P-V catheter in PAH rats versus control rats with or without LPS injection. We found that 1 mg/kg of LPS induced severe depression in LV contractility in PAH rats, whereas LV function in normal rats remained at baseline levels. We can explain the occurrence and metastasis mechanisms of this phenomenon in terms of two facts. First, LPS induced acute right heart failure in rats with PAH. Our previous study demonstrated that 1 mg/kg of LPS induced acute right heart failure in rats with PAH [4]. However, it was reported that 6 mg/kg of LPS induced non-lethal sepsis in rats and that 5 hours after LPS treatment, LV ejection fraction decreased more significantly in the PAH group than in the control group [14]. The same dosage of LPS was proven to depress cardiomyocyte sarcomere shortening and to result in LV ejection fraction depression in mice [15]. We can conclude that PAH rats were more susceptible to low-dose LPS-induced inflammation damage. Second, right heart failure can damage left heart function and eventually lead to whole heart failure. It was reported that increments of capacity overload and pressure overload of the RV in rats with PAH caused LV dysfunction [11]. This ventricular interdependence could most likely explain why the RV affected the LV [16], and the progress of leftward septal shift caused further LV dysfunction [17]. In our study, we used the P-V catheter to measure ventricular function and ventricular-vascular coupling, independent of loading conditions [18]. The P-V catheter is not only a tool for achieving a clinical standard diagnosis for PAH but also can be used in small animals [19]. As P-V catheter data showed, heart rate, cardiac output, ejection fraction, dP/dt_max⁡_, and stroke work in PAH rats significantly decreased after LPS treatment, whereas Tau increased. The results indicate that acute inflammation leads to dysfunction of the LV. The plunge of the ejection fraction and stroke work in PAH rats after LPS injection suggests that an effective treatment for acute heart failure is necessary.

LPS depresses cardiomyocyte sarcomere shortening but inhibits sarcoplasmic reticulum Ca^2+^ ATPase function by reducing calcium uptake [20]. Enzyme-soluble guanylyl cyclase *α*1 is associated with LV ejection fraction depression and attenuation of cardiomyocyte sarcomere shortening [21].

CNP binds to the natriuretic peptide receptor, which induces the generation of cGMP [22]. Two predominant molecular forms of CNP in the porcine brain were reported to be a 22-residue peptide (CNP-22) and its N-terminally elongated 53-residue peptide (CNP-53). They induced cGMP production in a dose-dependent and similar fashion. It was suggested that CNP-53 could, in concert with other natriuretic peptides, have a neuromodulatory function and thereby contribute to the central regulation of hemodynamic and fluid homeostasis [23]. It has been reported that cGMP signaling plays an important role as an indicator of cardiac stress responses such as *β*-adrenergic stimulation, ischemic injury, and pressure and volume overload [24]. However, contradictory evidence has been revealed from models of experimental PAH and patients with PAH [25]. In our study, an infusion of CNP ameliorates LPS-induced cardiac dysfunction in PAH rats. The protective effects of CNP mainly result from a reduction in the afterload and a positive inotropic effect. The reduction in the afterload is due to natriuretic peptide effects on cGMP to an activation of a particulate guanylyl cyclase [26]. CNP could stimulate the release of cGMP by activating natriuretic peptide receptor-B [27]. In our study, CNP treatment increased plasma cGMP levels in PAH rats that were injected with LPS. We propose that the active mechanism of CNP in our model was through the protein kinase G signaling pathway. The previous research suggested that cGMP accumulation was mediated by activation of certain kinases and not by protein kinase C, increased intracellular calcium, or other second messenger pathways, or protein phosphatases [23]. This pathological mechanism may be not activated in normal rats. In contrast, CNP activates natriuretic peptide receptor-B and cGMP release, which increases the production of protein kinase G, which is a very important molecule that activates sarcomere movement as well as sarcoplasmic reticulum function.

In this study, cGMP-mediated positive inotropic and relaxant effects were similar to those seen in a previous study [28]. cGMP has been reported to increase positive inotropic responses mediated by *β*1-adrenoceptor through inhibition of phosphodiesterase-3 [29]. It is reported that cGMP may increase intracellular cyclic adenosine 3′,5′-monophosphate, improve contractility, and restore cardiac function by regulating phosphodiesterase subtypes [30]. This study may improve the understanding of the functional implications and compartmentalization of cGMP.

A limitation of this study might be that we did not investigate whether CNP could improve the prognosis of severe PAH. The molecular mechanisms of protein kinase G signaling should be further investigated also.

In conclusion, CNP could salvage LPS-induced cardiac dysfunction and restore deterioration hemodynamics in PAH rats. This effect may be related to cGMP signaling regulation.

## Figures and Tables

**Table 1:** Cardiac morphometric data.

**Parameters**	**Control group**	**PAH group**
	**(n = 10)**	**(n = 10)**
BW (g)	428.6 ± 37.18	375.7 ± 29.06*∗∗*
**HW (g)**	1.06 ± 0.05	1.13 ± 0.22
**RV weight (g)**	0.25 ± 0.03	0.43 ± 0.15*∗∗*
**RV/(LV + S) weight**	0.3 ± 0.04	0.61 ± 0.19*∗∗*
**mPAP (mmHg)**	5.66 ± 0.72	57.78 ± 10.55*∗∗*

Values are expressed as means ± SD; BW: body weight; HW: heart weight; RV: right ventricle; LV: left ventricle; S: septum; mPAP: mean pulmonary pressure. *∗P *< 0.05: compared with control group. *∗∗P *< 0.01: compared with control group.

**Table 2:** Effects of LPS on hemodynamics and left ventricle function.

Parameters	C group	C + LPS group	PAH group	PAH + LPS group
HR (bpm)	405.09 ± 20.89	398.93 ± 18.3	312.83 ± 31.28	285.64 ± 4.94*∗∗*
ESP (mmHg)	119.55 ± 10.14	120.78 ± 8.01	105.55 ± 16.15	85.93 ± 10.1*∗∗*
EDP (mmHg)	3.22 ± 2.61	1.1 ± 2.55	0.84 ± 2.17	3.69 ± 0.33*∗∗*
ESV (mL)	44.72 ± 12.62	48.46 ± 16.81	134.64 ± 45.03	81.49 ± 6.67*∗∗*
EDV (mL)	139.69 ± 18.85	139.69 ± 18.85	215.45 ± 63.13	106.08 ± 3.58*∗∗*
CO (mL)	43.14 ± 5.01	43.33 ± 6.41	26.48 ± 7.33	9.93 ± 1.23*∗∗*
EF (%)	75.73 ± 9.2	74.48 ± 9.03	39.9 ± 9.52	33.3 ± 4.77*∗*
dP/dt_max⁡_ (mmHg/s)	11508.2 ± 4000.4	11800.3 ± 3345.6	9183.1 ± 1456.6	6439.7 ± 930.2*∗*
-dP/dt_min⁡_ (mmHg/s)	10137.2 ± 1170.9	10220.1 ± 1603.1	7371.4 ± 1700.6	6320.3 ± 880.0*∗*
SW (mmHg × mL)	12106.0 ± 1900.5	12376.0 ± 2396.5	7901.4 ± 2360.5	2553.2 ± 202.3*∗∗*
Tau (ms)	8.13 ± 0.87	7.98 ± 0.68	10.29 ± 0.96	11.86 ± 0.43*∗∗*

Values are presented as mean ± SD, n = 10 in each group. C group: control group; HR: heart rate (beats/min); ESP: end systolic pressure; EDP: end diastolic pressure; ESV: end systolic volume; EDV: end diastolic volume; CO: cardiac output (mL/min); EF: ejection fraction (%); dP/dt_max⁡_: maximum rate of rise of LV pressure (mmHg/s); -dP/dt_min⁡_: minimum rate of rise of LV pressure (mmHg/s); SW: stroke work (mmHg × mL); Tau: time constant of relaxation (ms). *∗P *< 0.05: when compared with PAH group. *∗∗P *< 0.01: when compared with PAH group.

**Table 3:** Hemodynamic parameters and the indices of LV function.

**Parameters**	**Group**	**T0**	**T1**	**T2**
HR (bpm)	P1	353.46 ± 18.5	339.74 ± 21.24*∗*	308.82 ± 32.94*∗*
	P2	364.7 ± 23.27	318.13 ± 11.63*∗*#	285.64 ± 4.94*∗*#
	P3	349.78 ± 12.84	316.71 ± 12.90*∗*#	328.19 ± 29.91#$
ESP (mmHg)	P1	117.63 ± 15.57	108.3 ± 12.36	105.55 ± 16.15
	P2	124.75 ± 5.96	95.24 ± 6.29*∗*#	85.93 ± 10.1*∗*#
	P3	123.03 ± 7.12	106.43 ± 16.31*∗*#	105.90 ± 15.14*∗*$
EDV (*μ*L)	P1	203.49 ± 92.09	259.44 ± 79.5	219.03 ± 60.86
	P2	190.74 ± 85.14	115.08 ± 9.68*∗*#	106.08 ± 3.58*∗*#
	P3	266.06 ± 100.41	131.29 ± 26.81*∗*#	187.92 ± 68.02$
**SV (** ***μ*** **L)**	P1	100.9 ± 23.75	110.44 ± 27.71	93.39 ± 31.63
	P2	99.7 ± 23.75	71.34 ± 35.74*∗*#	34.75 ± 4.16*∗*#
	P3	111.72 ± 18.54	55.47 ± 7.93*∗*#	83.12 ± 28.63*∗*$
CO (mL)	P1	35.78 ± 8.82	33.74 ± 6.95	28.01 ± 7.21*∗*
	P2	37.38 ± 6.76	17.10 ± 1.97*∗*#	9.93 ± 1.23*∗*#
	P3	27.92 ± 1.19	17.53 ± 2.45*∗*#	39.06 ± 6.41*∗*$
EF (%)	P1	60.46 ± 15.44	54.82 ± 3.65*∗*	43.74 ± 9.76*∗*
	P2	61.67 ± 13.93	44.38 ± 4.45*∗*#	33.3 ± 4.77*∗*#
	P3	57.88 ± 16.32	42.54 ± 4.31*∗*#	46.61 ± 4.48$
dP/dt_max⁡_	P1	10072.6 ± 1131.9	9458.3 ± 1210.7	9183.1 ± 1456.5*∗*
	P2	10922.6 ± 3081.6	7808.7 ± 1188.0*∗*#	6439.7 ± 930.2*∗*#
	P3	10567.2 ± 1013.5	8612.1 ± 1972.4*∗*#	8839.8 ± 1674.1*∗*$
SW	P1	11153.1 ± 2472.0	10245. 0 ± 2913.3	12119.7 ± 2288.4*∗*
	P2	9935.1 ± 2301.56	5445.4 ± 2853.2*∗*#	5299.7 ± 1397.82#
	P3	12119.7 ± 2288.4	5299.7 ± 1397.8*∗*#	7829.7 ± 2916.2*∗*$
Tau	P1	9.16 ± 1.34	9.81 ± 1.1*∗*	10.43 ± 0.38*∗*
	P2	9.24 ± 1.49	10.78 ± 0.84*∗*#	11.86 ± 0.43*∗*#
	P3	9.15 ± 1.31	10.21 ± 0.58*∗*#	8.8 ± 1.31#$

Values were presented as mean ± standard deviation. Group P1: PAH rats treated with normal saline. Group P2: PAH rats treated with LPS. Group P3: PAH rats treated with LPS and CNP. T0: baseline parameters. T1: 1 hour after treatment with normal saline (P1) and LPS (P2, P3). T2: 1 hour after sequential treatment with normal saline (P1, P2) and CNP (P3). HR: heart rate (beats/min); ESP: end systolic pressure; EDV: end diastolic volume; SV: stroke volume (mL); CO: cardiac output (mL/min); EF: ejection fraction (%); dP/dt: rate of rise of LV pressure (mmHg/s); SW: stroke work (mmHg × ml); Tau: time constant of relaxation (ms). *∗P *< 0.05: compared with T0 in each group, #*P *< 0.05: compared with P1 at same time point and $*P* < 0.05: compared with P2 at same time point.

## Data Availability

The data used to support the findings of this study are included within the article. The datasets analyzed during the current study are available from the corresponding author on reasonable request.

## Abbreviations

PAH: Pulmonary arterial hypertension
CNP: C-type natriuretic peptide
LPS: Lipopolysaccharide
P-V: Pressure-volume
RV: Right ventricular
LV: Left ventricular
S: Septum
dP/dt max: Rate of LV pressure rise in early systole
dV/dt max: Peak positive rate of mitral flow
Tau: Time constant of relaxation
ELISA: Enzyme-linked immunosorbent assay
cGMP: Cyclic guanosine 3′,5′-monophosphate.
